# Prophylactic A-Blockers for Radiotherapy-Induced Lower Urinary Tract Symptoms in Men with Prostate Cancer: A Phase III Randomized Trial

**DOI:** 10.3390/cancers15133444

**Published:** 2023-06-30

**Authors:** Tamim Niazi, Edmond Kaldany, Steven Tisseverasinghe, Talía Malagón, Boris Bahoric, Victor McPherson, Alexis Rompre-Brodeur, Maurice Anidjar

**Affiliations:** 1 Division of Radiation Oncology, Department of Oncology, McGill University, Montreal, QC H3A 0G4, Canada; 2 Division of Radiation Oncology, Department of Oncology, McGill University, Gatineau, QC J8V 3R2, Canada; 3 Division of Cancer Epidemiology, Department of Oncology, McGill University, Montreal, QC H3A 0G4, Canada; 4 Division of Urology, Department of Surgery, McGill University, Montreal, QC H3A 0G4, Canada

**Keywords:** prostate cancer, prophylactic alpha-blockers, lower urinary tract symptoms, radiation therapy

## Abstract

**Simple Summary:**

Prostate cancer radiation therapy (RT) to the pelvis is one of the curative approaches for patients with localized prostate cancer. Most patients treated with prostate RT experience some degree of radiation-induced lower urinary tract symptoms (RI-LUTSs). These symptoms may have a negative impact on patients’ quality of life after treatment. The aim of our prospective study was to determine if prophylactic α-blockers improved RI-LUTSs in men undergoing prostate RT. Overall, we found that there were no important effects of prophylactic α-blockers on RI-LUTSs. Therefore, treating prostate cancer patients preventatively with α-blockers may cause additional drug toxicity and financial burden without significantly improving their urinary symptoms. This trial was registered with ClinicalTrials.gov under the registration number NCT02220829.

**Abstract:**

Purpose: The present phase III randomized trial assessed the efficacy of prophylactic versus therapeutic α-blockers at improving RI-LUTSs in prostate cancer patients receiving external beam radiotherapy (EBRT). Methods: A total of 148 prostate cancer patients were randomized 1:1 to receive either prophylactic silodosin on day one of EBRT or the occurrence of RI-LUTSs. LUTSs were quantified using the international prostate symptom score (IPSS) at regular intervals during the study. The primary endpoint was the change in the IPSS from baseline to the last day of radiotherapy (RT). Secondary endpoints included changes in IPSS from baseline to 4 weeks and 12 weeks after the start of RT. Results: Patient demographics, baseline IPSS, and prescribed radiation doses were balanced between arms. On the last day of RT, the mean IPSS was 14.8 (SD 7.6) in the experimental arm and 15.7 (SD 8.5) in the control arm (*p* = 0.40). There were no significant differences in IPSSs between the study arms in the intention-to-treat (ITT) analysis at baseline, the last day of RT, and 4 and 12 weeks post-RT. Conclusion: Prophylactic α-blockers were not effective at significantly reducing RI-LUTSs in prostate cancer patients treated with EBRT. Treating patients with α-blockers at the onset of RI-LUTSs will avoid unnecessary drug exposure and toxicity.

## 1. Introduction

Prostate cancer is the second most common cancer among men worldwide [1]. In the United States alone, it is estimated that 288,300 men will be diagnosed with prostate cancer and 34,700 men will die of it in 2023 [2]. Early-stage prostate cancer patients are often offered radiation therapy (RT) with or without a high-dose-rate brachytherapy (HDR-BT) boost as primary treatment. Patients who undergo external beam radiation therapy (EBRT) can experience radiation-induced lower urinary tract symptoms (RI-LUTSs) [3]. This is due to radiation’s potential to cause direct urothelial damage along with inflammation of the prostate stroma, bladder, and urethral mucosa [4]. The resulting LUTSs include storage symptoms, such as nocturia, frequency, urgency, and urge incontinence, or voiding symptoms, such as incomplete voiding or weaker urinary flow. LUTSs seldom require the suspension or discontinuation of treatment, but they can significantly affect patients’ quality of life [3]. The severity of the symptoms may be partly affected by the dose of radiation delivered, the treatment technique used, as well as the number of fractions prescribed [4]. 

RI-LUTSs are commonly managed by administering α-blockers which act by inhibiting the activity of α-adrenergic receptors in the urethra and bladder neck. These receptors are G-protein coupled receptors (GPCRs) that, when stimulated by a catecholamine, induce the contraction of smooth muscle fibers. Although these receptors exist as three subtypes (α-1A, -1B, and -1D), α-1A adrenoceptors are the primary mediators of smooth muscle tone in the bladder, prostate, and urethra [5,6,7]. Therefore, by antagonizing the α-1A receptors and reducing smooth muscle contraction around the prostate, α-1 blockers can improve urinary flow and thus relieve LUTSs. 

Presently, several α-blockers are available and have demonstrated variable selectivity for different α-1 adrenoceptor subtypes [8,9]. Among these, tamsulosin has been the mainstay for combatting RI-LUTSs [10,11]. Tamsulosin antagonizes both α-1A and -1D receptors. Alternatively, silodosin (Rapaflo) acts more selectively on α-1A receptors. The preferential blocking of the α-1A receptor has been theorized to minimize cardiovascular side effects [12,13]. In 2011, Japanese investigators compared the efficacy of prophylactic naftopidil versus tamsulosin and silodosin in prostate cancer patients undergoing low-dose-rate (LDR) BT with iodine-125 seed implants. The trial demonstrated a reduction in the LUTSs, favoring patients using silodosin [14]. Similarly, Merrick et al. also showed a significant improvement in the recovery from urinary morbidities for prostate cancer patients treated with LDR BT [15]. 

Given the lack of prospective clinical trials, we designed a phase III randomized controlled trial (RCT) to determine whether prophylactic α-1-blockers would decrease the rate and intensity of LUTSs, improve their recovery post-EBRT and maximize patients’ quality of life during and after RT. 

## 2. Methods

### 2.1. Trial Conduct

The trial was conducted at the Jewish General Hospital located in Montreal, Quebec, Canada. The trial was approved by the institutional ethics review board and was conducted in compliance with the guidelines for the Good Clinical Practice of the International Conference on Harmonization and the principles of the Declaration of Helsinki. All the patients provided written informed consent prior to their enrollment. 

### 2.2. Patients and Trial Regimen

Eligible patients were 18 years of age or older with a confirmed diagnosis of localized adenocarcinoma of the prostate, treated primarily with EBRT with or without HDR BT boost. Patients with a history of radical prostatectomy, metastatic (M1), or locally advanced (T4) disease invading surrounding organs in the pelvic cavity, severe hepatic (Child–Pugh score ≥ 10) or renal impairment (CCr < 30 mL/min) were excluded from the study. 

The computer-generated randomization process was conducted centrally. Upon successful enrolment into the trial, treatment allocation was performed in an unblinded manner. Patients received 8 mg of silodosin PO daily either prophylactically or therapeutically upon symptom onset only. Patients randomized in the prophylactic α-blocker group would take the medication starting on Day 1 of RT for a total duration of six months: 8 mg daily for four months, and then 8 mg every other day for the last two months. 

### 2.3. Assessments

During the screening period, patient demographics and relevant medical history were gathered. Blood samples were collected for the assessment of liver function (alkaline phosphatase, alanine transaminase, bilirubin conjugate, and total) and kidney function (urea, creatinine). In addition, initial LUTSs were evaluated through the IPSS questionnaire, which was subsequently repeated at pre-specified time points: four weeks after the start of RT, on the last day of RT, and monthly thereafter for six months. The IPSS questionnaire was also repeated at the nine- and twelve-month marks after the start of RT. While post-void residual urine tests were recommended during and after RT if indicated by the treating physician, they were not mandatory. 

### 2.4. Questionnaire

The IPSS is a modified version of the American Urological Association symptoms index (AUA-SI) [16]. It contains seven questions on the frequency and intensity of different LUTSs, each graded from 0 to 5 based on severity. The combination of these seven subscores will result in an overall score ranging between 0 and 35. The questionnaire also has an independent QOL query that does not factor in the total IPSS index. 

### 2.5. Study Outcomes 

The primary outcome was a change in mean IPSS from baseline to the last day of RT. Secondary outcomes included a change in mean IPSS from baseline to 4 weeks and 12 weeks post-RT initiation, as well as the rate of return to baseline 3 months post-treatment.

### 2.6. Statistical Analysis

To assess the difference in the pre-specified outcomes between study arms, we fitted a linear regression model to all participants’ IPSS measures during the course of the trial. The main analysis was an intention-to-treat (ITT) analysis, with the study arm included as a predictor in the model. The model also included time since randomization as a categorical predictor and an interaction term in order to assess the interaction between the study arm and time. The correlation between repeated IPSS measures on the same participant was accounted for by assuming an unstructured covariance structure across observations, estimated using residual restricted maximum likelihood and empirical (“sandwich”) estimators. This covariance structure was selected because IPSS scores are expected to increase during the trial and then return to baseline levels over time, so other covariance structures assuming an equal or declining correlation over time were inappropriate for this analysis. Differences in the IPSS scores between arms and over time were estimated using model coefficients. The significance of predictor variables in the model was assessed using t and F statistics. 

We also performed an as-treated analysis, comparing all patients who received prophylactic α-blockers to those who did not receive medication prophylactically (either did not receive medication or received it at the onset of RI-LUTSs). For this analysis, we refitted the linear regression model using treatment received as a predictor instead of the study arm.

In another exploratory analysis, we fitted a linear model to assess the association between the receipt of an α-blocker prescription and IPSS scores over time in the control arm. Due to the smaller number of observations in this analysis, a heterogeneous compound symmetry covariance structure was used to model within-subject correlation. All statistical analyses were performed using SAS software, version 9.4 (Copyright © 2013 SAS Institute Inc. SAS and all other SAS Institute Inc. product or service names are registered trademarks or trademarks of SAS Institute Inc., Cary, NC, USA.). 

## 3. Results

### 3.1. Patient Accrual and Baseline Features

**Figure 1:**
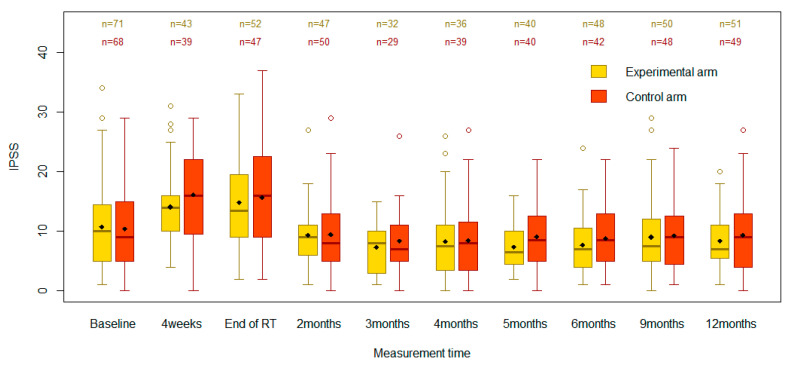
Boxplots comparing IPSSs between the control and experimental groups across the specified time points.

In total, 148 participants were recruited from January 2016 to November 2020, with 921 observations included in the analysis (Figure 1). There was a large variation in IPSS scores across individuals. The participants were well balanced across study arms for all demographics and most clinical features (Table 1). A slightly higher proportion of participants received whole-pelvis radiotherapy versus prostate-only radiotherapy in the control arm (49%) relative to the experimental arm (40%). All participants in the experimental arm group received prophylactic α-blockers. Twenty-seven participants (38%) in the control arm group received a therapeutic prescription for α-blockers, and four participants (6%) deviated from the protocol by receiving prophylactic α-blockers pre-BT. All participants who were prescribed an HDR BT boost received a single fraction of 15 Gy, in addition to EBRT. 

### 3.2. Primary and Secondary Endpoints

At baseline, the mean IPSS was 10.8 (standard deviation [SD] 7.3) in the experimental arm and 10.4 (SD 7.2) in the control arm (Table 2). There were no statistically significant differences between the two groups at baseline (*p* = 0.77). The scores increased in both the experimental and control groups to 14.8 (SD 7.6) and 15.7 (SD 8.5), respectively, by the end of RT. These changes were important, as assessed by a type III test for the effect of time (*p* < 0.0001). However, there were no significant differences between the two groups at the end of RT (*p* = 0.40). The IPSS increase from baseline to RT end was smaller in the experimental arm than in the control arm, but the −1.3 difference in score change from baseline was not significant (95%CI −4.5 to 1.8, *p* = 0.40). The as-treated analysis showed similar results (Table 3), with the change in IPSS from baseline to RT end being not significantly different between the groups (*p* = 0.13). 

At 4 weeks post-RT start, in the ITT analysis, the mean IPSSs rose to 14.1 (SD 6.2) and 16.1 (SD 8.0) in the experimental and control groups, respectively (Table 2). However, there were no statistically significant differences between the study groups (95%CI −5.4 to 0.7, *p* = 0.13). In both groups, the mean IPSS declined after the end of RT. While the 3-month mean IPSSs appeared lower than baseline in both arms, the confidence intervals overlapped (*p* = 0.17). Based on the type III test of fixed effects, the arm effect (*p* = 0.28) and the arm-by-time interaction effect (*p* = 0.84) were not statistically significant in the ITT analysis, suggesting there was no evidence that the prophylactic intervention had a different effect than standard care on the evolution of IPSS scores over time.

Overall, the as-treated analysis yielded nearly similar results to the ITT analysis with the IPSS increasing from baseline within each group at 4 weeks post-RT start and RT end and returning close to baseline as early as three months after treatment initiation. However, a single important difference of −3.4 in IPSS at 4 weeks post-RT start (95%CI −5.6 to 0.0, *p* = 0.03) in favor of the arm receiving prophylactic α-blockers was identified (Table 3). On average, participants receiving prophylactic α-blockers noted a smaller increase from baseline IPSS at 4 weeks than those who did not, with score changes of 3.3 (95%CI 1.3 to 5.2) and 6.7 (95%CI 4.4 to 9.0), respectively. While a type III test of fixed effects showed a significant time effect (*p* < 0.0001), the group effect (*p* = 0.29) and the group-by-time interaction effect (*p* = 0.84) were not significant. 

### 3.3. Subgroup Analysis of Control Arm

A subgroup analysis limited to the control arm (N = 76) was performed (Table 4). The mean IPSS was higher at nearly all time points for control patients who received α-blockers at any point during the study timeline (*n* = 31) compared to those who did not (*n* = 45). However, these differences were not statistically significant in the overall type III test for the medication effects (*p* = 0.09). 

### 3.4. Exploratory Analyses

Exploratory analyses, whereby participants were stratified per prostate volume (PV) and T-stage, were also conducted. In an as-treated analysis restricted to participants with PVs <50 cc, a difference of −4.3 between groups in favor of prophylaxis was identified at 4 weeks post-RT initiation (95%CI −8.4 to −0.3) (Table 5). Hence, the IPSS surge from baseline was more pronounced in the control group relative to the prophylactic group of patients with PVs <50 cc at 4 weeks into RT. This difference was not observed in the as-treated analysis limited to participants with larger PVs. In the ITT and as-treated analyses, respectively, where participants were stratified per T-stage, the IPSSs were equivalent across arms in both low- (*p* = 0.49, *p* = 0.47) and high-stage settings (*p* = 0.54, *p* = 0.54) at all time points.

## 4. Discussion

RI-LUTSs can arise during and following prostate RT [17]. Most patients recover from acute urinary toxicity within a few months after treatment [17,18]. Accordingly, in our trial, mean IPSSs increased during and shortly after RT in the ITT analysis, regardless of the study arm. LUTSs improved and returned to baseline levels as early as three months post-RT for both the experimental and control groups. The change in mean IPSS over time was highly significant for each study arm. Yet, the prophylactic medication had no perceptible effects on the participants’ IPSSs. There were no statistically important differences in mean IPSSs between the study groups at all time points in the ITT analysis. Overall, the fluctuations in IPSSs were equivalent across time points for the two study groups. These results imply that the participants likely experienced similar levels of urinary toxicity during and post-RT regardless of the study arm and that the prophylactic use of α-blockers had no measurable effect on the recovery rate.

Although α-blockers are known to help alleviate LUTSs in patients undergoing BT, there have been no dedicated retrospective or prospective studies on its prophylactic use during prostate EBRT to our knowledge. Merrick et al. found that administering prophylactic α-blockers prior to LDR-BT can mitigate LUTSs [15]. Furthermore, in an RCT evaluating tamsulosin in BT patients, Elshaikh et al. found significantly decreased LUTSs limited to week 5 in the experimental arm [19]. The authors attributed this to the resolution of prostate edema, unrelated to the α-blockers.

Our as-treated analysis yielded similar results to the ITT analysis, finding no significant difference in changes in IPSSs between the two groups at all time points except at 4 weeks into RT in favor of the treatment arm. Should it not be due to chance, this sole significant result may be clinically relevant [16,20]. Similarly, the exploratory as-treated analysis restricted to participants with PVs inferior to 50 cc showed a statistically and clinically significant difference at 4 weeks in favor of prophylactic α-blockers. Despite this, an overall type III test for the time-specific effect of prophylactic α-blockers was not significant (*p* = 0.25). Therefore, the four-week results could be attributable to chance from multiple testing rather than a consequence of prophylactic medication use. In the event that this result was not due to chance, it could suggest that prophylactic α-blockers were temporarily beneficial for LUTSs during RT in men with PVs <50 cc, similar to other published data [21,22,23]. In their prospective clinical trial, Matsukawa et al. concluded that silodosin improved storage functions, such as detrusor overactivity and bladder capacity, in men with prostate with ≤40 cc over 2 years relative to men with larger PVs [23]. Furthermore, α-blockers have been reported to alter collagen fibers in the prostatic stroma [24]. This phenomenon may be more prominent in men with smaller PVs, resulting in a greater loss of smooth muscle around the prostate. Alternatively, α-blockers can modulate bladder blood flow (BBF), an important player in improving storage function [23].

If we exclude the participants who received prophylactic α-blockers pre-BT, about 60% of patients analyzed in Arm 2 did not receive a therapeutic prescription for silodosin at any point during or after RT. Prescribing α-blockers to all patients undergoing RT may unnecessarily expose them to unwarranted drug toxicity and financial burden. In fact, depending on the prescribed medication, adverse effects can include retrograde ejaculation, dizziness, or orthostatic hypotension [25,26].

Our study has some limitations. After performing a post hoc power calculation for a two-sided two-sample t-test on paired differences, we concluded the study would have a 20% chance to detect a difference of 1.3 with an α-level of 0.05. The low power is due to the effect size being small relative to the standard deviations in IPSS scores between participants. However, our study had an 80% chance to detect an IPSS difference of 3.3 with the same α-level. The Measurement Committee of the American Urological Association (AUA) defined the minimal clinically important difference (MCID) for an improvement of LUTSs as a −3.1 difference in IPSS [16,20]. In our trial, the participants experienced overall mild-to-moderate LUTSs at baseline. Based on the post hoc power calculation and available MCID values, our trial has a reasonable statistical power to detect a clinically important difference in IPSS between the two groups despite the small sample size.

Furthermore, the assessment of medication adherence was not ideal. Patients self-reported compliance during follow-ups. While refills were also monitored through medical records, the pills were not manually counted. Hence, a self-reporting bias may exist. While there is no gold standard for measuring adherence, the combination of two corroborative methods may have been more reliable [27].

## 5. Conclusions

Our study demonstrates that prophylactic silodosin administration does not significantly decrease RI-LUTSs in patients receiving EBRT for prostate cancer. We, therefore, recommend initiating therapeutic α-blockers upon symptom onset. Consequently, this practice would improve efficacy, avoid treatment futility in the preventative setting, and spare prostate cancer patients from unnecessary toxicities.

## Figures and Tables

**Table 1:** Patient baseline characteristics and treatment received.

	Experimental Arm (Prophylaxis) *n* = 76		Control Arm (Standard Care) *n* = 72	
	Mean	SD	Mean	SD
Age at baseline (years)	73.0	6.6	70.7	6.6
Prostate volume (cc)	51.6	28.3	43.4	22.9
	N	(%)	N	(%)
**Race**				
White	59	78%	59	82%
Black	8	11%	6	8%
Other	4	5%	4	6%
Missing	5	6%	3	4%
**T stage**				
Low risk (T1c, cT1c, T2a, cT2a, rT2a)	43	57%	38	53%
Intermediate risk (T2b, T2b/T3a, T2b/T3b, T2c, T2c/T3a, cT2c)	13	17%	16	22%
High risk (T3a, T3a/b, T3b, cT3a, rT3a, rT3b, T4a)	20	26%	18	25%
**Gleason score**				
6	5	7%	0	0%
7	48	63%	48	67%
8	11	14%	14	19%
9	9	12%	10	14%
10	3	4%	0	0%
**Urinary incontinence**				
No	66	87%	65	90%
Yes	4	5%	6	8%
Missing	6	8%	1	2%
**Urinary frequency/urgency**				
No	59	78%	50	70%
Yes	11	14%	21	29%
Missing	6	8%	1	1%
**Hematuria**				
No	70	92%	71	99%
Missing	6	8%	1	1%
**Dysuria**				
No	66	87%	68	94%
Yes	4	5%	2	3%
Missing	6	8%	2	3%
**Other baseline toxicities**				
No	28	37%	29	40%
Yes	42	55%	42	58%
Missing	6	8%	1	2%
**RT dose**				
<66 Gy	4	5%	4	6%
66/68 Gy	21	28%	20	28%
76 Gy	30	39%	31	43%
78 Gy	18	24%	17	23%
>78 Gy	1	1%	0	0%
None	2	3%	0	0%
**Number of fractions**				
<22	1	1%	0	0%
22	20	26%	19	26%
>22–<38	4	5%	5	7%
38	31	41%	31	43%
39	18	24%	17	24%
None	2	3%	0	0%
**Treatment Technique Prescribed**				
IMRT	72	95%	72	100%
Unknown/No Data	4	5%	0	0%
**Volume Irradiated**				
Whole-Pelvis	30	40%	35	49%
Prostate-Only	42	55%	36	50%
Unknown/No Data	4	5%	1	1%
**Brachy Boost**				
No	74	97%	68	94%
Yes	2	3%	4	6%
**Received alpha-blockers**				
No	0	0%	41	57%
Yes—therapeutic	0	0%	27	38%
Yes—prophylactic	76	100%	4	6%

RT = radiotherapy.

**Table 2:** Intention to treat analysis: IPSS mean measures over time, model-estimated differences between arms, differences from baseline within the arm, and differences between arm baseline differences.

	Mean IPSS (SD)				Model Estimates								
Time	Experimental Arm (*n* = 76)		Control Arm (*n* = 72)		Difference in Arm Means (95%CI)		Baseline Difference, Experimental Arm (95%CI)		Baseline Difference, Control Arm (95%CI)		Difference of Arm Baseline Differences (95%CI)		*p*-Value
Baseline	10.8	(7.3)	10.4	(7.2)	0.4	(−2.1 to 2.8)	Ref	-	Ref	-	-	-	-
4 weeks	14.1	(6.2)	16.1	(8.0)	−2.0	(−4.7 to 0.8)	3.7	(1.8 to 5.6)	6.0	(3.7 to 8.4)	−2.3	(−5.4 to 0.7)	0.13
End of RT	14.8	(7.6)	15.7	(8.5)	−1.0	(−3.9 to 2.0)	4.1	(1.9 to 6.4)	5.5	(3.2 to 7.8)	−1.3	(−4.5 to 1.8)	0.40
2 months	9.3	(4.8)	9.5	(6.2)	−0.6	(−2.7 to 1.4)	−1.5	(−3.3 to 0.4)	−0.5	(−2.2 to 1.3)	−1.0	(−3.5 to 1.6)	0.45
3 months	7.3	(4.1)	8.3	(5.4)	−1.7	(−3.7 to 0.3)	−3.4	(−5.4 to −1.4)	−1.3	(−3.5 to 0.8)	−2.1	(−5.0 to 0.9)	0.17
4 months	8.3	(6.1)	8.4	(6.1)	−0.4	(−2.8 to 2.0)	−2.5	(−4.8 to −0.1)	−1.7	(−3.6 to 0.2)	−0.8	(−3.7 to 2.2)	0.62
5 months	7.3	(4.0)	9.1	(5.7)	−1.3	(−3.2 to 0.6)	−3.0	(−4.2 to −1.4)	−1.3	(−3.3 to 0.7)	−1.7	(−4.2 to 0.8)	0.19
6 months	7.7	(4.5)	8.8	(5.1)	−0.9	(−2.8 to 0.9)	−2.8	(−4.2 to −1.4)	−1.5	(−3.5 to 0.4)	−1.3	(−3.7 to 1.1)	0.29
9 months	9.0	(6.2)	9.3	(5.7)	−0.1	(−2.3 to 2.0)	−1.7	(−3.3 to 0.0)	−1.2	(−2.7 to 0.4)	−0.5	(−2.8 to 1.8)	0.66
12 months	8.4	(4.3)	9.4	(6.3)	−0.6	(−2.6 to 1.3)	−2.0	(−3.5 to −0.4)	−1.0	(−2.6 to 0.6)	−1.0	(3.2 to 1.2)	0.37

**Table 3:** As-treated sensitivity analysis: IPSS mean measures over time, with model-estimated differences between arms, differences from baseline within the arm, and differences between arm baseline differences.

	Mean IPSS (SD)				Model Estimates								
Time	Prophylactic (*n* = 80)		Non-Prophylactic (*n* = 68)		Difference in Group Means (95%CI)		Baseline Difference, Prophylactic (95%CI)		Baseline Difference, Non-Prophylactic (95%CI)		Difference of Group Baseline Differences (95%CI)		*p*-Value
Baseline	10.9	(7.5)	10.2	(7.0)	0.6	(−1.8 to 3.0)	Ref	-	Ref	-	-	-	-
4 weeks	13.8	(6.2)	16.6	(7.9)	−2.8	(−5.6 to 0.0)	3.3	(1.3 to 5.2)	6.7	(4.4 to 9.0)	−3.4	(−6.4 to −0.4)	0.03
End of RT	14.6	(7.6)	16.0	(8.6)	−1.8	(−4.8 to 1.1)	3.7	(1.5 to 5.9)	6.1	(3.9 to 8.4)	−2.4	(−5.6 to 0.7)	0.13
2 months	9.2	(4.7)	9.7	(6.3)	−0.7	(−2.8 to 1.4)	−1.6	(−3.4 to 0.2)	−0.2	(−2.0 to 1.5)	−1.3	(−3.9 to 1.2)	0.29
3 months	7.3	(4.1)	8.3	(5.4)	−1.5	(−3.5 to 0.6)	−3.4	(−5.4 to −1.4)	−1.3	(−3.5 to 0.9)	−2.1	(−5.0 to 0.9)	0.16
4 months	8.3	(6.0)	8.5	(6.2)	−0.7	(−3.1 to 1.5)	−2.7	(−4.9 to −0.5)	−1.4	(−3.3 to 0.6)	−1.3	(−4.3 to 1.6)	0.37
5 months	7.5	(4.2)	9.0	(5.7)	−0.7	(−2.6 to 1.2)	−2.8	(−4.4 to −1.1)	−1.4	(−3.4 to 0.5)	−1.3	(−3.8 to 1.2)	0.31
6 months	8.0	(4.9)	8.5	(4.7)	−0.5	(−2.3 to 1.4)	−2.7	(−4.2 to −1.2)	−1.6	(−3.5 to 0.5)	−1.1	(−3.5 to 1.3)	0.37
9 months	9.2	(6.3)	9.0	(5.6)	0.3	(−1.8 to 2.4)	−1.6	(−3.2 to 0.1)	−1.2	(2.8 to 0.3)	−0.3	(−2.6 to 1.9)	0.78
12 months	8.5	(4.7)	9.3	(6.0)	−0.6	(−2.6 to 1.4)	−2.0	(−3.6 to −0.4)	−0.8	(−2.4 to 0.7)	−1.2	(−3.4 to 1.1)	0.30

**Table 4:** Control arm subgroup analysis: IPSS mean measures over time, with model-estimated differences between arms, differences from baseline within the arm, and differences between arm baseline differences.

	Mean IPSS (SD)				Model Estimates								
Time	Received Alpha-Blockers (*n* = 31)		Did Not Receive Alpha-Blockers (*n* = 45)		Difference in Group Means (95%CI)		Baseline Difference, Received LUTSs Medication (95%CI)		Baseline Difference, No LUTSs Medication (95%CI)		Difference of Group Baseline Differences (95%CI)		*p*-Value
Baseline	11.2	(8.2)	9.7	(6.3)	1.6	(−1.9 to 5.1)	Ref	-	Ref	-	-	-	-
4 weeks	18.9	(7.6)	12.9	(7.4)	4.2	(−0.2 to 8.6)	7.0	(3.2 to 10.7)	4.3	(1.6 to 7.1)	2.7	(−2.0 to 7.3)	0.26
End of RT	17.3	(8.8)	13.4	(7.9)	2.9	(−1.5 to 7.3)	6.2	(2.9 to 9.5)	4.9	(1.6 to 8.2)	1.4	(−3.3 to 6.0)	0.56
2 months	9.1	(6.4)	9.8	(6.1)	−0.2	(−3.4 to 3.1)	−1.2	(−3.8 to 1.3)	0.5	(−1.9 to 2.8)	−1.7	(−5.2 to 1.7)	0.32
3 months	9.8	(6.2)	6.3	(3.3)	3.1	(0.0 to 6.1)	−1.1	(−4.7 to 2.5)	−2.6	(−5.1 to −0.1)	1.5	(−2.8 to 5.9)	0.49
4 months	9.2	(7.1)	7.6	(4.9)	1.3	(−1.9 to 4.5)	−2.0	(−5.2 to 1.2)	−1.8	(−4.0 to 0.5)	−0.2	(−4.2 to 3.7)	0.90
5 months	9.6	(5.8)	8.3	(5.6)	2.0	(−1.3 to 5.2)	−1.2	(−4.3 to 1.8)	−1.6	(−4.5 to 1.2)	0.4	(−3.8 to 4.6)	0.85
6 months	9.2	(5.9)	8.2	(3.5)	1.6	(−1.1 to 4.2)	−1.7	(−4.9 to 1.5)	−1.7	(−4.0 to 0.5)	0.0	(−3.9 to 3.9)	1.00
9 months	10.4	(6.5)	7.8	(4.3)	3.2	(0.3 to 6.1)	−0.5	(−3.1 to 2.0)	−2.2	(−4.1 to −0.3)	1.6	(−1.5 to 4.8)	0.30
12 months	9.8	(7.0)	8.8	(5.3)	1.3	(−2.0 to 4.5)	−1.1	(−3.6 to 1.4)	−0.8	(−3.0 to 1.4)	−0.3	(−3.6 to 3.1)	0.86

**Table 5:** As-treated sensitivity analysis in men with prostate volume less than 50 (cc): IPSS mean measures over time, with model-estimated differences between arms, differences from baseline within the arm, and differences between arm baseline differences.

Prostate Volume Less than 50 (cc)							
	Mean IPSS(SD)			Model Estimates			
	Prophylactic	Non	Difference in Group	Baseline Difference	Baseline Difference	Difference of Group	*p*-Value
		Prophylactic	Means	Prophylactic	Non Prophylactic	Baseline Difference	
			(95%CI)	(95%CI)	(95%CI)	(95%CI)	
Baseline	10.3 (8.3)	9.5 (7.2)	0.8 (−2.8 to 4.4)	-	-	-	-
4 weeks	11.7 (5.9)	16.0 (8.0)	−3.5 (−7.2 to 0.1)	2.4 (−0.5 to 5.4)	6.8 (4.0 to 9.6)	−4.3 (−8.4 to −0.3)	0.04
End of RT	13.4 (8.0)	16.8 (8.4)	−3.5 (−7.4 to 0.5)	3.4 (0.1 to 6.6)	7.6 (4.6 to 10.7)	−4.3 (−8.7 to 0.2)	0.06
2 months	8.1 (4.0)	10.1 (7.0)	−1.3 (−4.3 to 1.6)	−1.5 (−4.3 to 1.3)	0.6 (−1.8 to 3.0)	−2.1 (−5.8 to 1.5)	0.26
3 months	7.3 (3.5)	8.3 (5.6)	0.0 (−2.9 to 3.0)	−1.6 (−4.5 to 1.2)	−0.9 (−3.9 to 2.2)	−0.8 (−4.9 to 3.4)	0.72
4 months	8 (7.0)	7.7 (5.4)	−0.1 (−3.3 to 3.2)	−2.1 (−5.9 to 1.6)	−1.3 (−3.8 to 1.3)	−0.9 (−5.4 to 3.6)	0.70
5 months	7.4 (3.9)	8.6 (5.5)	−0.5 (−3.1 to 2.1)	−2.5 (−5.2 to 0.3)	−1.1 (−3.8 to 1.5)	−1.3 (−5.2 to 2.5)	0.50
6 months	8.1 (5.6)	7.3 (3.3)	0.9 (−1.4 to 3.3)	−2.2 (−4.4 to 0.0)	−2.4 (−4.8 to 0.1)	0.1 (−3.2 to 3.4)	0.93
9 months	9.7 (7.3)	8.9 (6.1)	1.0 (−2.1 to 4.1)	−0.6 (−2.9 to 1.7)	−0.8 (−2.9 to 1.3)	0.2 (−2.9 to 3.3)	0.91
12 months	8.7 (5.1)	9.4 (6.5)	−0.6 (−3.3 to 2.2)	−1.5 (−4.0 to 0.9)	−0.2 (−2.2 to 1.9)	−1.4 (−4.6 to 1.8)	0.40

## Data Availability

The data presented in this study are available on request from the corresponding author. The data are not publicly available due to privacy concerns.
